# Effect of *trans*(NO, OH)-[RuFT(Cl)(OH)NO](PF_6_) ruthenium nitrosyl complex on methicillin-resistant *Staphylococcus epidermidis*

**DOI:** 10.1038/s41598-019-41222-0

**Published:** 2019-03-19

**Authors:** Mathilde Bocé, Marine Tassé, Sonia Mallet-Ladeira, Flavien Pillet, Charlotte Da Silva, Patricia Vicendo, Pascal G. Lacroix, Isabelle Malfant, Marie-Pierre Rols

**Affiliations:** 1 Laboratoire de Chimie de Coordination du CNRS, 205 route de Narbonne, F-31077 Toulouse, France; 2 Institut de Pharmacologie et de Biologie Structurale, IPBS, Université de Toulouse, CNRS, UPS, Toulouse, France; 3 Laboratoire des Interactions Moléculaires et de la Réactivité Chimique et Photochimique, Université Paul Sabatier, 118 route de Narbonne, F-31062 Toulouse, France

**Keywords:** Chemical biology, Photochemistry

## Abstract

Antibiotic resistance is becoming a global scourge with 700,000 deaths each year and could cause up to 10 million deaths by 2050. As an example, *Staphylococcus epidermidis* has emerged as a causative agent of infections often associated with implanted medical devices. *S*. *epidermidis* can form biofilms, which contribute to its pathogenicity when present in intravascular devices. These staphylococci, embedded in the biofilm matrix, are resistant to methicillin, which had long been the recommended therapy and which has nowadays been replaced by less toxic and more stable therapeutic agents. Moreover, current reports indicate that 75 to 90% of *Staphylococcus epidermidis* isolates from nosocomial infections are methicillin-resistant strains. The challenge of successfully combating antibiotics resistance in biofilms requires the use of compounds with a controlled mode of action that can act in combination with antibiotics. Ruthenium nitrosyl complexes are potential systems for NO release triggered by light. The influence of *trans*(NO, OH)-[RuFT(Cl)(OH)NO](PF_6_) on *Staphylococcus epidermidis* resistant to methicillin is described. The results show a 50**%** decrease in cell viability in bacteria treated with low concentrations of NO. When combined with methicillin, this low dose of NO dramatically decreases bacterial resistance and makes bacteria 100-fold more sensitive to methicillin.

## Introduction

Antimicrobial resistance is a globally discerned problem, recognized as one of the greatest threats to health^1^. *Staphylococcus epidermidis* strains are often resistant to antibiotics, including rifamycin, fluoroquinolones, gentamicin, tetracycline, clindamycin, and sulfonamides. Methicillin resistance is particularly widespread, with 75–90% of hospital isolates resistant to methicillin. The adjective “methicillin-resistant” is used to characterize resistance to virtually all β-lactams (except to latest generation cephalosporins)^2,3^. Moreover, studies recently showed that three lineages of *Staphylococcus epidermidis* have developed a resistance against rifampicin in numerous countries. This indicates that hospital practices have driven the evolution of this organism, once trivialized as a contaminant, towards potentially incurable infections^4^. Aggregated communities of bacteria, such as the ones present in biofilms, increase bacterial tolerance to hazardous environments and antibiotics^5^. Increased antimicrobial tolerance in biofilms is responsible for chronic infections and failures of antibiotic therapies^6^. While being inefficient to control biofilms expansion, exposure to sub-inhibitory concentrations of many antibiotics can facilitate biofilm formation. The biofilm matrix is a niche that favors the appearance of resistance, inhibits the penetration of antibiotics and prevents antibiotics to reach biofilm-embedded cells^7^. Studies indicate that killing bacteria in a biofilm may require up to 1000 times the antibiotic dose, which would be necessary to achieve the same result in a suspension of cells. Biofilm formation is reported as a key virulence factor in microorganisms that cause chronic infections^8^. The nature of biofilm development and drug tolerance implies great challenges in the use of conventional antimicrobials, and indicates the need for multi-targeted or combination therapies including phototherapies^9^. Biofilm-targeting technologies aimed at disrupting the complex biofilm microenvironment^10^ and thus inducing the liberation of planktonic susceptible bacteria are indeed a clinical necessity. Recently, a simple gas, which is also a ubiquitous biological signaling molecule, the nitric oxide (NO), was identified as a key mediator of biofilm dispersal occurring across microbial species^6^. NO has therefore great potential for novel therapeutics. In addition, inhaled NO gas was approved as therapeutic agent by FDA in 1999. Since then, it has been used as pulmonary vasodilator in pulmonary hypertension treatment^11^. A combined treatment of low dose (500 nM) of NO^•^ gas with intravenous administration of ceftadizime and tobramycin has been used for the eradication of *P*. *aeruginosa* biofilms in cystic fibrosis patients^12^. At the opposite, high concentrations of NO^•^ (in the millimolar range) can have undesirable effects. At high doses NO can be toxic to surrounding tissues and can inhibit wound healing because of its immunosuppressant properties. High levels of NO^•^ can also induce defense mechanisms in bacteria, rendering them more tolerant to antibiotics^6^. Moreover, a study showed that exposure to millimolar concentration of NO^•^ can trigger a response from the biofilm, leading to its increased formation^13^.

In this context, exogenous NO· donors are widely investigated, but their relevance has to be evaluated based on their ability to deliver NO· locally and quantitatively, in order to avoid undesirable effects on untargeted cells. Among potential candidates, ruthenium-nitrosyl complexes have been recognized as the most promising candidates^14–17^, in relation to their generally low toxicity, good stability and capability of releasing NO· under light irradiation in the *λ* = 300–600 nm range, exclusively taking advantage of the non-invasive and highly controllable characteristics of light. Although the photochemical pathway is not yet completely characterized, the NO· release can be generally described by the following reaction:

$$[{{\rm{Ru}}}^{{\rm{II}}}{({\rm{NO}})}^{+}]+{\rm{solvent}}\mathop{\longrightarrow }\limits^{{\rm{hv}}}[{{\rm{Ru}}}^{{\rm{III}}}({\rm{solvent}})]+{{\rm{NO}}}^{\bullet }$$

Previous studies on parent *cis*(Cl, Cl)- and *trans*(Cl, Cl)-[RuFTCl_2_NO](PF_6_) complexes with 4′-(2-fluorenyl)-2,2′:6′,2″-terpyridine (FT) have demonstrated their efficiencies in NO photo-delivery upon one-photon excitation at 405 nm, as well as upon two-photon excitation in the NIR region. Moreover, cytotoxicity and phototoxicity studies have provided evidence showing that these complexes are efficient candidates, that could serve as photoactivatable molecular tools for resection of malignancies^18,19^ or bactericidal agent. Therefore, the *trans*(NO, OH)-[RuFT(Cl)(OH)NO](PF_6_) complex derived from the previous systems is a relevant candidate for acting on resistant bacteria.

Efficiency studies on bacterial cells are generally performed during planktonic growth, yet bacterial natural habitats often include communities disseminated within biofilms, which are characterized by dramatically different physiological properties. During the past decades, there has been a consensus around the development of a biofilm model, involving attachment of single planktonic bacterial cells to a surface and the subsequent development of a mature biofilm. Recent data show that bacterial aggregates perform better than single cells and, over long time scales, biofilm structures are likely to become dominated by progeny originating from preformed aggregates^20^. As in biofilms, bacteria in aggregates are protected. In contrast to biofilms, however, metabolic activity is high in aggregates. Aggregates provide bacteria with the benefits of a biofilm while maintaining mobility. This combination contributes to the difficulties of eradicating bacteria, which become highly resistant to antibiotic treatments^5^.

In this work, we have examined a type of community, namely cellular aggregates, observed in human pathogenic strains such as *Staphylococcus epidermidis* ATCC 35984, which is resistant to methicillin and responsible of nosocomial infections, as a proof of concept to test the influence of the NO photo-release from *trans*(NO, OH)-[RuFT(Cl)(OH)NO](PF_6_) on the recovery of the susceptibility of the bacteria towards the antibiotic.

## Results and Discussion

### Characteristics of NO release by irradiation of *trans*(NO, OH)-[RuFT(Cl)(OH)NO](PF_6_)

**Figure 1:**
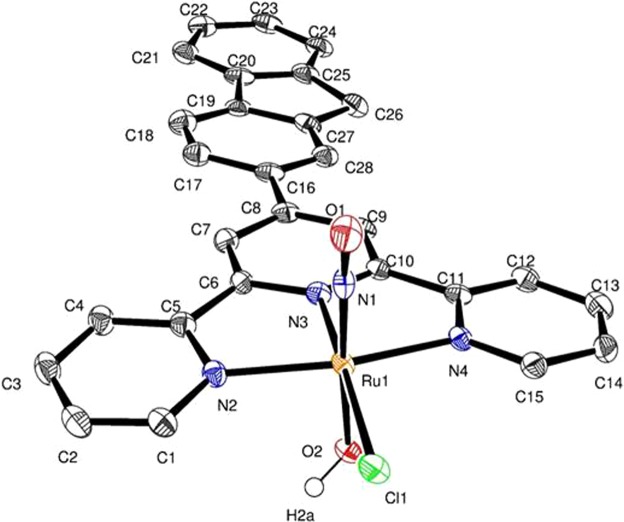
*Trans*(NO, OH)-[RuFT(Cl)(OH)NO]^+^ complex. Displacement ellipsoids are drawn at the 50% probability level. Hydrogen atoms are omitted for clarity except H2a from OH ligand.

The search for alternative NO· donors led to the study of a new complex *trans*(NO, OH)-[RuFT(Cl)(OH)NO](PF_6_). The complex is synthesized from previously described^18^ *trans*(Cl, Cl)-[RuFTCl_2_NO](PF_6_) in water (ESI). Single crystals, suitable for X-ray determination, were obtained from diffusion of diethyl ether in acetonitrile solution of complexes (ESI). The structure of the cationic complex is shown on Fig. 1. The data are in agreement with the well-known {RuNO}^6^ Enemark configuration^14^ described here as [Ru^II^(NO)^+^].

**Figure 2:**
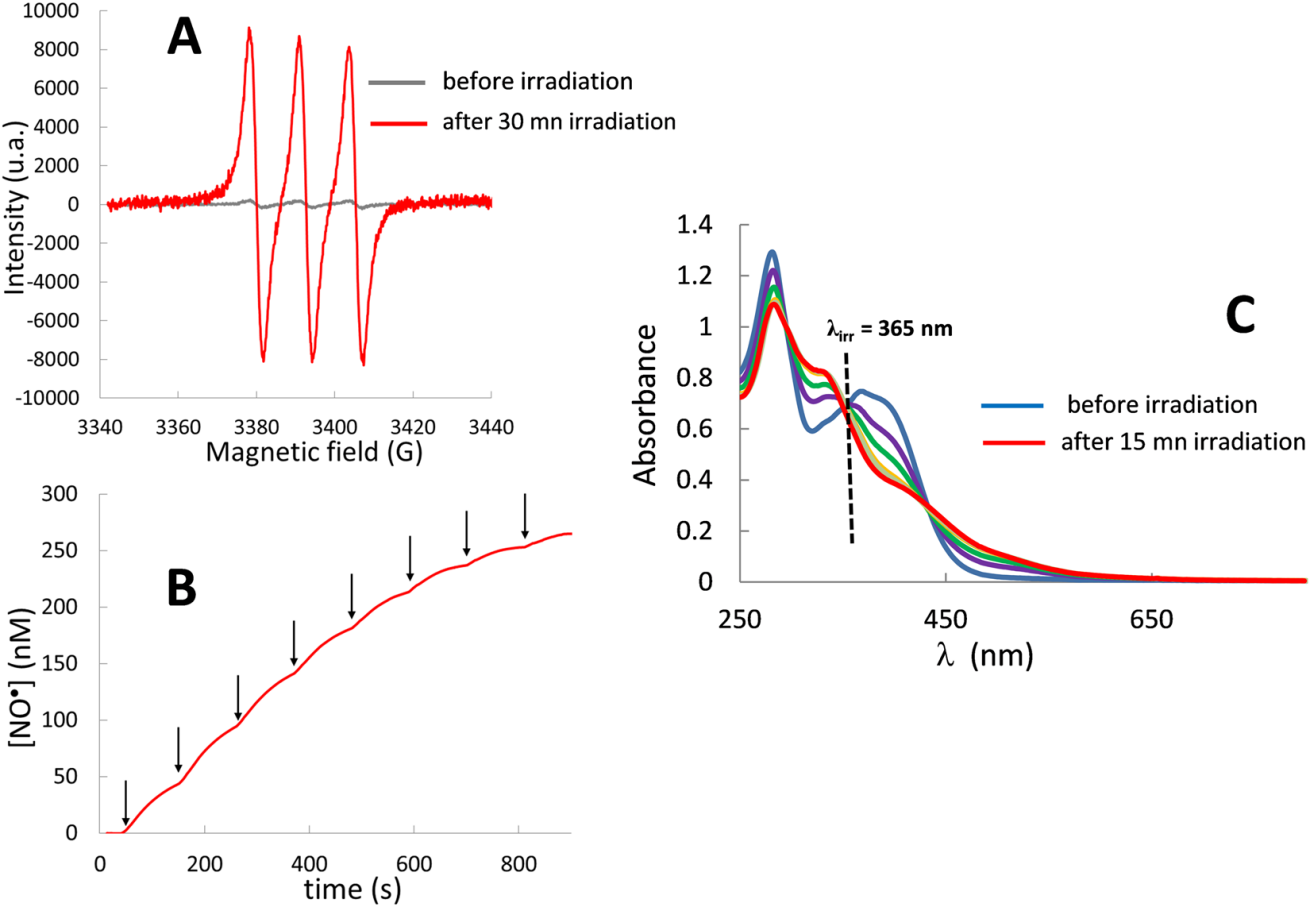
NO photorelease from *trans*(NO, OH)-[RuFT(Cl)(OH)NO](PF_6_). Triplet electron paramagnetic resonance signals from NO trapping by [Fe(MGD)_2_] (**A**); Chronoamperograms of NO upon irradiation steps of 15 s (arrows). The typical sensitivity of the NO detector was about 100 pA/nM (**B**); Evolution of absorption spectra of *trans*(NO, OH)-[RuFT(Cl)(OH)NO](PF_6_) in water (0.5% DMSO) at 365 nm. (**C**).

The NO photo-release is demonstrated by using EPR spectroscopy, since spin trapping combined with EPR spectroscopy is considered as one of the best methods for the direct detection of NO· radicals^21^. We used Iron(II)-N-methyl-D-glucamine dithiocarbamate [Fe^II^(MGD)_2_] to trap NO due to the high probability of adduct formation and to the high stability of its spin adduct. A solution of *trans*(NO, OH)-[RuFT(Cl)(OH)NO](PF_6_) in water (0.5% DMSO) was analyzed under one photon irradiation using a mercury lamp. Figure 2A shows the characteristic triplet signal with a hyperfine splitting constant value of a_N_ = 1.2 10^−3^ cm^−1^ and a *g*-factor of 2.040. This is consistent with the literature report for [Fe^II^(MGD)_2_-NO] adduct^22^.

Moreover, direct NO release was confirmed by NO sensor measurements. The chronoamperogram of *trans*(NO, OH)-[RuFT(Cl)(OH)NO](PF_6_) shows a NO· concentration up to 265 nM (Fig. 2B).

The quantum yield of the complex can be determined from the evolution of its absorption spectrum under irradiation and was carried out at 365 nm with monochromatic LEDs.

The changes in the electronic absorption spectra exposed to 365 nm light in water (0.5% DMSO) are shown in Fig. 2C. The presence of isosbestic points at 346 and 426 nm indicates a clean conversion of the Ru(II)(NO) complex to the related photolysed species. The quantum yield of *trans*(NO, OH)-[RuFT(Cl)(OH)NO](PF_6_) at 365 nm is 0.040 with *ε*_365_ = 16333 L. mol^−1^. cm^−1^ (ESI). This value is a relevant parameter to evaluate the NO release as each NO radical is delivered from the reaction of the former complex.

### Characteristics of the bacterial strains

**Figure 3:**
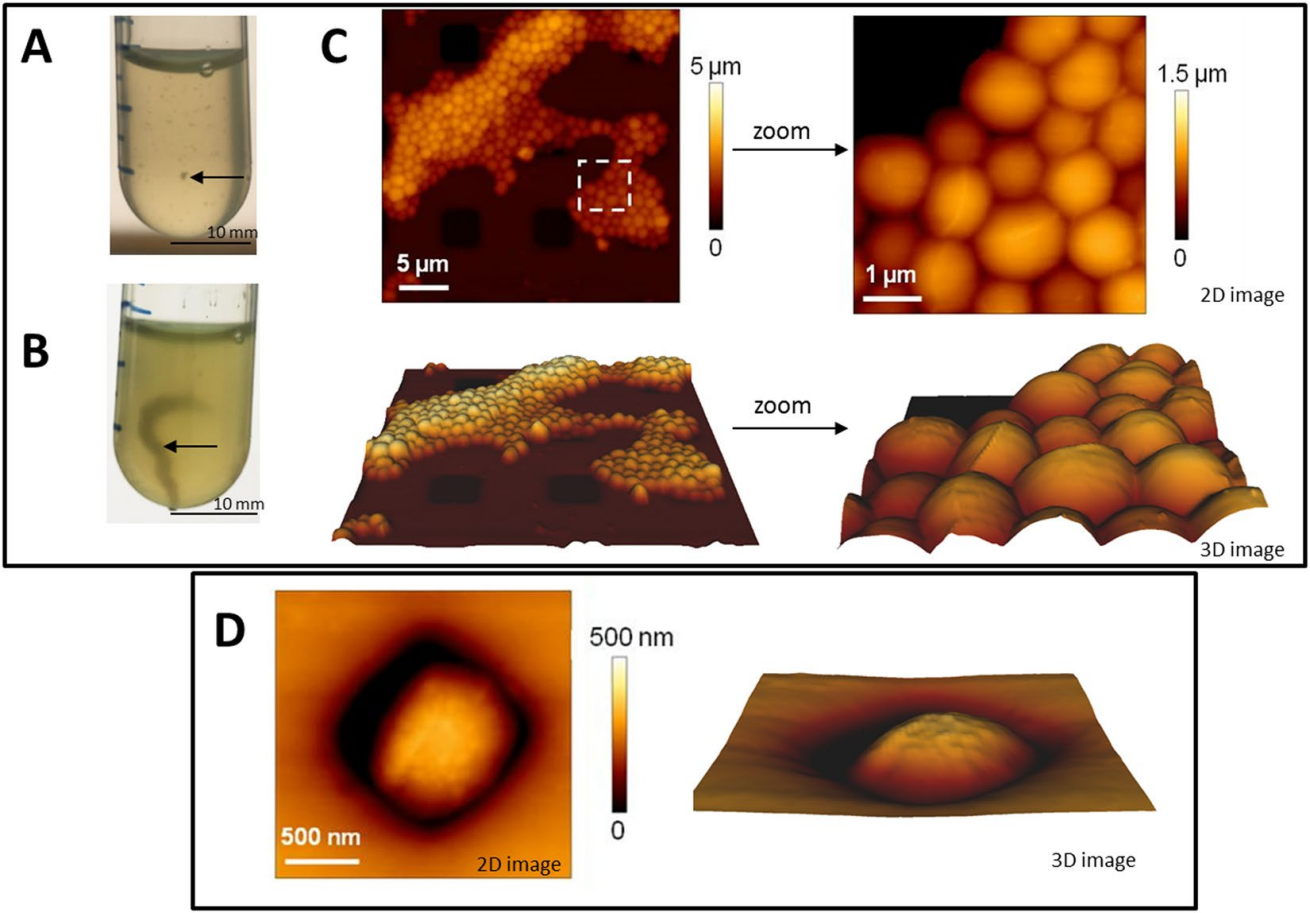
Observation of *S*. *epidermidis* ATCC 35984 and ATCC 12228. *S*. *epidermidis* ATCC 35984 after 3 hours (**A**) or 10 hours (**B**) of culture under agitation in LB (arrows show aggregates and filaments). 2D or 3D AFM images of *S*. *epidermidis* ATCC 35984 (**C**) and *S*. *epidermidis* ATCC 12228 (**D**).

Two strains of *Staphylococcus epidermidis* have been used in this work: (i) *S*. *epidermidis* ATCC 35984 known to be resistant to methicillin and to form biofilms^23–26^ and (ii) *S*. *epidermidis* ATCC 12228 known to be sensitive to the antibiotic and not form biofilms^27^. As shown in Fig. 3, the ATCC 35984 strain formed aggregates, visible to the naked eye after 3 hours of culture (Fig. 3A). These aggregates further formed large filaments after 10 hours (Fig. 3B), providing evidence of the ability of the strain to form a biofilm, which under the conditions of culture under agitation did not attach to the bottom of the tube.

Atomic force microscopy was used to study the morphology and the nano-mechanical properties of living microorganisms^28,29^. As shown in Fig. 3C, the *S*. *epidermidis* ATCC 35984 strain forms aggregates of hundreds of cells. On the contrary, the bacteria of the *S*. *epidermidis* ATCC 12228 strain are isolated (Fig. 3D). A mean roughness of 2.8 ± 1.7 nm was determined for the sensitive bacteria and 5.6 ± 5.9 nm for the antibiotic resistant ones. Mechanical properties were determined by successive force measurements. For sensitive bacteria, the stiffness was 152 ± 29 mN/m. For resistant bacteria, a decrease in this value and in the homogeneity of the distribution of values was observed, with a mean of 98 ± 52 mN/m. These differences may reflect the presence of the extra cellular matrix^30^, leading to the change in the organization of the cell wall surface and to the organization of the bacteria into biofilm. *Staphylococcus epidermidis* can therefore be present and cultivated under different forms: planktonic and aggregates with different mechanical properties.

### Bactericide effect of NO from *trans*(NO, OH)-[RuFT(Cl)(OH)NO](PF_6_)

Bacteria were grown to exponential growth phase. Under this condition, the *S*. *epidermidis* ATCC 35984 strain forms aggregates and the *S*. *epidermidis* ATCC 12228 remains in the form of planktonic, individualized cells. Neither the presence of 0.5% of DMSO (necessary to solubilize *trans*(NO, OH)-[RuFT(Cl)(OH)NO](PF_6_)) nor irradiation up to 10 minutes had any effect on bacteria growth (ESI). The generation time remained close to 35 minutes, a value in agreement with the values found in the literature^31^.

Increasing concentrations of *trans*(NO, OH)-[RuFT(Cl)(OH) NO](PF_6_) from 0.05 to 1 µM were added to the cells. Concentrations above 1 µM inhibited cell growth, and affected cell viability, as determined by colony counting (ESI). Indeed, *trans*(NO,OH)-[RuFT(Cl)(OH)NO](PF_6_) had no toxic effect for concentrations up to 0.5 µM. Above 1 µM, it induced a 40% decrease in viability even in the absence of irradiation. Under irradiation, the toxicity could be observed already at 0.1 µM, with a 2 fold decrease in cell viability. The same was observed for the *S*. *epidermidis* ATCC 12228 strain (ESI). *Trans*(NO, OH)-[RuFT(Cl)(OH)NO](PF_6_) used at concentrations below 1 µM did not result in total eradication of bacteria. Higher concentrations are needed to eradicate bacteria but are also toxic to tissues, as they hamper wound healing (NO· limits inflammation and reduces macrophage activity).

### Combination of NO with methicillin

**Figure 4:**
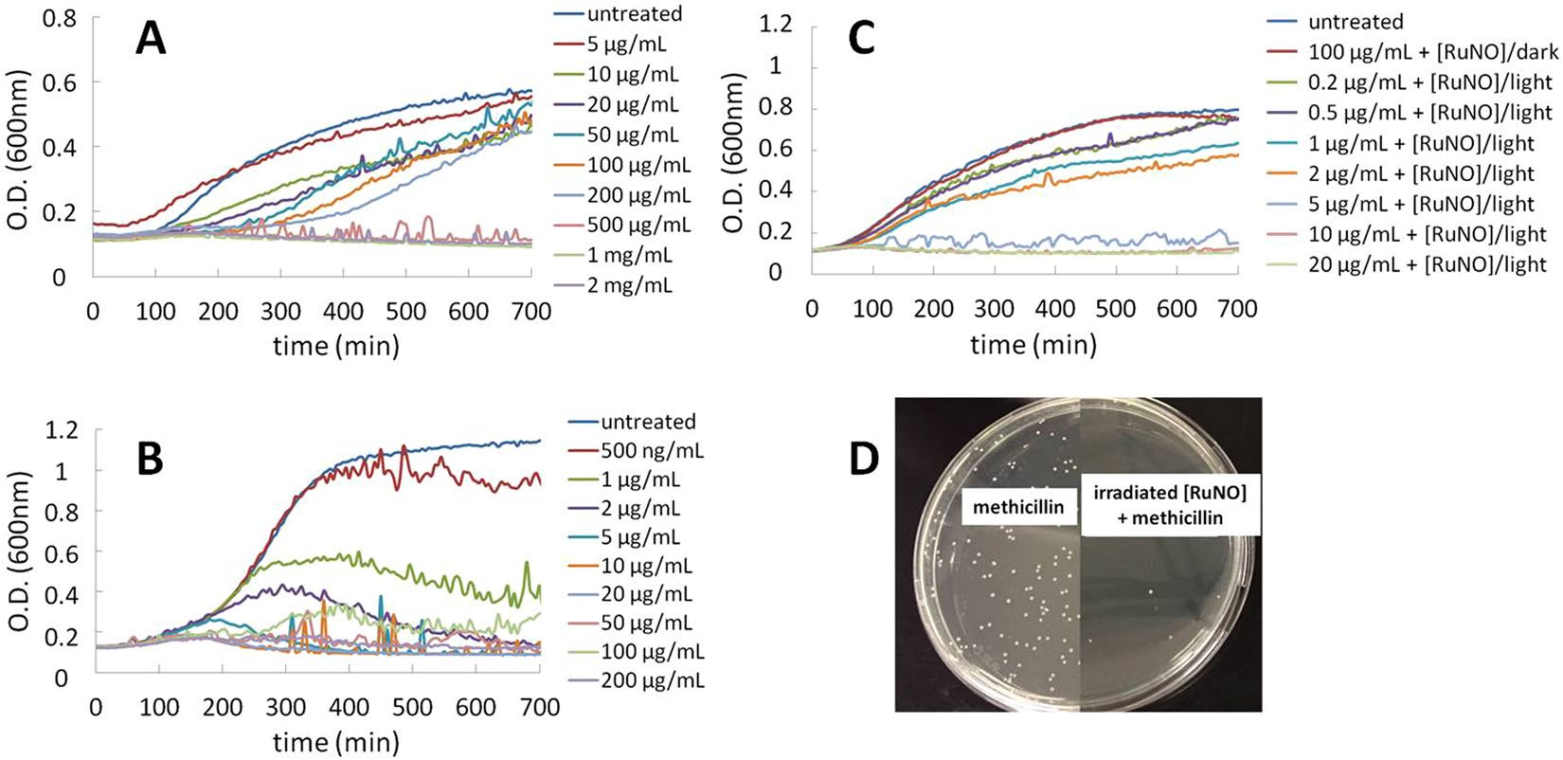
Growth of *Staphylococcus epidermidis*: Effect of methicillin on *S*. *epidermidis* ATCC 35984 (**A**) and *S*. *epidermidis* ATCC 12228 (**B**). Effect of methicillin and *trans*(NO, OH)-[RuFT(Cl)(OH)NO](PF_6_) on *S*. *epidermidis* ATCC 35984. (**C**) Without/with irradiation of 0.1 µM [RuNO]; (**D**) Number of colonies of *S*. *epidermidis* ATCC 35984 after treatment with methicillin (left side) and with combined treatment ([RuNO]-methicillin (right side) in presence of irradiated [RuNO]. [RuNO] stands for *trans*(NO, OH)-[RuFT(Cl)(OH)NO](PF_6_) and was irradiated during 10 minutes with a Hg lamp (32 mW).

Therefore, we investigated an alternative strategy, based on the combination of a low dose of *trans*(NO, OH)-[RuFT(Cl)(OH)NO](PF_6_), applied simultaneously with antibiotics. The ATCC 12228 strain, which is sensitive to antibiotics, and the *S*. *epidermidis* ATCC 35984 strain resistant to methicillin have been cultivated in the presence of different concentrations of methicillin (from 0.5 µg/mL to 200 µg/mL for *S*. *epidermidis* ATCC 12228 and from 5 µg/mL to 2 mg/mL for *S*. *epidermidis* ATCC 35984). The choice of these concentrations ranges was based on previously published works^32^. MIC of 500 µg/mL (Fig. 4A) and 5 µg/mL (Fig. 4B) have been determined for the resistant and the sensitive strain respectively in LB. This 2 log difference is in agreement with the fact that one strain is resistant to methicillin.

The effect of the combined action of a low dose of *trans*(NO, OH)-[RuFT(Cl)(OH)NO](PF_6_) irradiated with the addition of the antibiotic was evaluated to determine if the resistant strain can become sensitive to the antibiotic. For *S*. *epidermidis* ATCC 35984, low methicillin concentrations (0.2 µg/mL to 20 µg/mL) were used.

The irradiation of antibiotic resistant bacteria with 0.1 µM *trans*(NO, OH)-[RuFT(Cl)(OH)NO](PF_6_) had a dramatic effect on the MIC that dropped from 500 µg/mL to 5 µg/mL in LB (Fig. 4C) and from 1 mg/mL to 10 µg/mL in MHB (2% NaCl (wt/vol)) (ESI). Intriguingly, in both cases, the combined action of the antibiotic and the controlled release of NO allowed to decrease by a 2 log factor the MIC of the antibiotic. At the opposite and as expected, for the sensitive *S*. *epidermidis* ATCC 12228 strain, the combined action of *trans*(NO, OH)-[RuFT(Cl)(OH)NO](PF_6_) and methicillin had a very low effect on the MIC, that only decreased from 5 µg/mL to 2 µg/mL both in LB and MHB (2% NaCl (wt/vol)) (ESI).

These results suggest that, in the case of *S*. *epidermidis* ATCC 35984, NO^•^ can induce the biofilm dispersion. The dispersed bacteria can return to their planktonic phenotype and do not express any resistance anymore. Consequently, methicillin treatment allows their eradication. Taken together, these results provide evidence for the high potential of combined treatment of *trans*(NO, OH)-[RuFT(Cl)(OH)NO](PF_6_) and methicillin to overcome the resistance of *S*. *epidermidis* ATCC 35984 to the antibiotic (Fig. 4D).

In a last series of experiments, which were designed to confirm that this effect is due to NO· released from the irradiated *trans*(NO, OH)-[RuFT(Cl)(OH)NO](PF_6_), the toxicity of a solution after irradiation was assayed. The solution contained the photoproduct but also the NO· oxidation products, such as nitrites NO_2_^−^ and nitrates NO_3_^−^. Such solutions had no effect on *S*. *epidermidis* ATCC 35984. Furthermore, in the presence of 5 µg/mL of methicillin, the photoproduct is not toxic (ESI).

## Conclusion

This work provides a piece of evidence suggesting the interest to develop combined strategies to eradicate resistant bacterial communities, such as the ones present in aggregates. This is of high importance for clinical applications, where the tissue infected by bacteria has to be preserved, and therefore the use of high amounts of chemicals or physical tools is prohibited. By disrupting the extracellular matrix, NO· leads to the release of bacteria from aggregates and to the recovery of their susceptibility toward methicillin. Molecular mechanisms should now be elucidated and the potential of this method should be tested on clinical samples. The perspective of this strategy is very promising, as it can be aimed against mature biofilms and might be applied to different species of bacteria.

## Methods

### Material and Equipment

The solvents were analytical grade and used without further purification. Elemental analyses were performed at LCC with a Perkin Elmer 2400 serie II Instrument. ^1^H NMR spectra were obtained at 298 K in CD_3_CN as internal reference and were recorded on a Bruker Avance 300 or a Bruker Avance 400. Infrared spectra were recorded on a Perkin Elmer 1725 Spectrometer. LC/MS experiments were performed on a Thermo Scientific LCQ Fleet ion trap mass spectrometer from Thermo Fisher Scientific. For EPR and NO sensor experiments, the light source was a 250 W Oriel Hg lamp (Palaiseau, France). The light was passed through an Oriel WG 400 UV filter (Palaiseau, France, λ > 400 nm) and delivered *via* an optical fiber to the grid of the cavity. For photokinetics experiments, WheeLED Wavelength-Switchable LED Sources (MIGHTEX WLS-22-A) were used at 365 nm (WLS-LED-0365-2) and 420 nm (WLS-LED-0420-3). Electron paramagnetic resonance experiments (EPR) were performed on a Brucker ESP 500E spectrometer. The following setting was employed for the measurements: microwave power, 20 mW, field modulation amplitude, 0.1 mT; field modulation frequency, 100 kHz; microwave frequency, 9.686899 GHz. N–methyl-D-glucamine dithiocarbamate previously synthetized reacted with Mohr salts to get [Fe(MGD)_2_]^21^. 90 µL of 1 mM of *trans*(NO, OH)-[RuFT(Cl)(OH)NO](PF_6_) were mixed with 10 µL of a 20 mM aqueous solution of [Fe(MGD)_2_] and injected into quartz capillaries. Samples were irradiated directly in the EPR cavity.

### Synthesis

*trans*(Cl, Cl)- and *cis*(Cl, Cl)-[RuFTCl_2_NO](PF_6_) were synthesised as previously reported^18,19^.

*trans*(NO, OH)-[RuFT(Cl)(OH)NO](PF_6_). RMN ^1^H (400 MHz, CD_3_CN, 298 K): δ (ppm) 9,23 (2H, dd, *J* = 5,7 Hz, 1,4 Hz, H6 et H6″), 8,96 (2H, s, H3′ et H5′), 8,85 (2H, d, *J* = 8,2 Hz, H3 et H3″), 8,52 (2H, td, *J* = 7,8 Hz, 1,5 Hz, H4 et H4″), 8,40 (1H, s, H1f), 8,24-8,16 (2 H, m, H3f et H4f), 8,06-7,98 (3H, m, H5f, H5 et H5″), 7,95 (1H, se, OH), 7,72 (1H, d, *J* = 6,8 Hz, H8f), 7,55-7,45 (2H, m, H6f et H7f), 4,16 (2H, s, H9f). IR(ATR): ν_NO_ = 1894 cm^−1^. Mass (ESI): m/z = 581,2 for [M^+^]. Elemental analysis found: C, 50.32; H, 3,31; N, 8,36. C_28_H_20_ClN_4_O_2_RuPF_6_, H_2_O requires C, 50.20; H, 3,31; N, 8,36.

### Crystallographic data

Data were collected at low temperature (100(2) K) on a Bruker Kappa Apex II diffractometer equipped with a 30 W air-cooled microfocus, using MoKα radiation (λ = 0.71073 Å), and an Oxford Cryosystems Cryostream cooler device. Phi- and omega- scans were used for data collection. The structure was solved by intrinsic phasing method (SHELXT)^33^_._ All non-hydrogen atoms were refined anisotropically by means of least-squares procedures on F² with the aid of the program SHELXL^34^. All the hydrogen atoms were refined isotropically at calculated positions using a riding model.

CCDC 1853344 contains the supplementary crystallographic data for this paper. These data can be obtained free of charge from the Cambridge Crystallographic data center.

### NO calibration

The quantitative determination of NO production was performed with a commercial NO detector (ami-NO 700) from Innovative Instruments Inc. Calibration of the electrode in the range of 50–1000 nM was performed by generating NO according to the following reaction:

$$2\,{{{\rm{NO}}}_{2}}^{-}+4\,{{\rm{H}}}^{+}+2\,{{\rm{I}}}^{-}\to 2\,{{\rm{NO}}}^{\bullet }+{{\rm{I}}}_{2}+2\,{{\rm{H}}}_{2}{\rm{O}}$$

For each calibration, aliquots (80 μL) of aqueous NaNO_2_ (∼100 μM) were added to 20 mL of a 0.03 mol.L^−1^ solution of KI in 0.1 mol.L^−1^ H_2_SO_4_. Chronoamperograms were registered at a fixed temperature (25 °C) while stirring the solution in order to maintain a constant rate of oxidation of the produced NO at the electrode surface. The typical sensitivity of the electrode was about 100pA/nM. During the photolysis measurements, the NO sensor was positioned outside the light path. Besides, chronoamperograms of an aqueous solution were systematically registered upon irradiation in order to substract the light interference. Then, chronoamperograms were registered upon irradiation of 20 mL of an aqueous solution of each complex in steps of 15 s every 110 s in order to stabilize the intensity between each step.

### Photochemistry

Kinetic studies on the photolysis reactions were carried out with a diode array Hewlett Packart 8454A spectrophotometer. The optical fiber was fixed laterally from the cuvette. Absorption spectra were taken after each minute, in fast scan mode. The UV-visible spectra were recorded under irradiation realized with a Muller reactor device equipped with a cooling water filter and monochromatic LEDs (see above). The light intensity was determined by using ferrioxalate actinometer. The sample solutions were placed in a quartz cuvette of 1 cm path -length stirred continuously. The temperature was maintained at 25 °C during the whole experiment.

Quantum yield measurements: Light intensities were determined before each photolysis experiments by chemical actinometry procedure. The light intensity was determined by using ferrioxalate actinometer. The quantum yield (ϕ_A_) was determined by the program Sa3.3 written by D. Lavabre and V. Pimienta^35^. It allows the resolution of the differential equation $\frac{{\rm{d}}[{\rm{A}}]}{{\rm{dt}}}=-\,{{\rm{\Phi }}}_{A}\,{{\rm{I}}}_{{\rm{a}}}^{{\rm{A}}}=-\,{{\rm{\Phi }}}_{A}\,{{\rm{Abs}}}_{{\rm{A}}}^{{\rm{\lambda }}}{{\rm{I}}}_{0}{\rm{F}}$ where ${{\rm{I}}}_{{\rm{a}}}^{{\rm{A}}}$ is the intensity of the light absorbed by the precursor; F, the photokinetic factor $({\rm{F}}=\frac{(1-{10}^{-{{\rm{Abs}}}_{{\rm{Tot}}}^{{\rm{\lambda }}}})}{{{\rm{Abs}}}_{{\rm{Tot}}}^{{\rm{\lambda }}}});\,{{\rm{Abs}}}_{{\rm{A}}}^{{\rm{\lambda }}}$, the absorbance of the complex before irradiation; ${{\rm{Abs}}}_{{\rm{Tot}}}^{{\rm{\lambda }}}$, the total absorbance; I_0_, the incident intensity measured at 365 nm. The equation was fitted with the experimental data ${{\rm{Abs}}}_{{\rm{Tot}}}^{{\rm{\lambda }}}=f(t)$ and 2 parameters ϕ_A_ and ε_B_ (ε_B_ is the molar extinction coefficient measured at the end of the reaction). λ_obs_ was chosen because it corresponds to a large difference between molar extinction coefficient at the initial and final time of the photochemical reaction. Simulation and optimization procedures were performed by using numerical integration and a non-linear minimization algorithm for the fitting of the model to the experimental data^35,36^. The conditions for the quantum yield determination for *trans*(NO, OH)-[RuFT(Cl)(OH)NO](PF_6_) complex in aqueous solution at 25 °C are gathered in Table S5 (ESI). Moreover, evidence for the inert photolysis product during the experiments has been systematically checked.

### Cell culture

Vegetative *Staphylococcus epidermidis* strains (ATCC 35984 and ATCC 12228) were cultivated in LB broth (Sigma-Aldrich, France) at 37 °C under agitation at 200 rpm. Their growth was monitored by optical density (OD) measurements at 600 nm.

### Evaluation of the inactivation rate by *trans*(NO, OH)-[RuFT(Cl)(OH)NO](PF_6_)

Cells were grown in 13 ml polypropylene tubes containing 3 ml of LB up to exponential growth phase (OD of 0.3). *trans*(NO, OH)-[RuFT(Cl)(OH)NO](PF_6_) complexes were prepared by dilution in LB of a 2 mM stock solution in DMSO and were immediately added to the bacteria at different concentrations of 0.1, 1, 2 and 5 μmol/L. After a 30 min incubation at 37 °C, tubes were irradiated 10 min with a Hg lamp (32 mW = dose 19.2 J.cm^−1^) or just kept in the dark. OD measurements were performed every 30 minutes.

Quantification of the bactericide effect of *trans*(NO, OH)-[RuFT(Cl)(OH)NO](PF_6_) was performed by spreading 100 µL of the 10^−5^ diluted bacterial suspension on LB agar Petri dishes. Inactivation rate was evaluated by colony counting 16 to 24 hours later. The proportion of inactivation was given as the ratio between untreated and irradiated bacteria. For each inactivation rate calculated, 3 independent experiments were applied with a total of 9 Petri dishes per analysis. Statistical analyses were performed with the Student’s t test.

### Evaluation of the inactivation rate by methicillin

Methicillin (methicillin sodium salt; BCBR6817V) was provided from Sigma. Stock solution was prepared by adding 500 μL of steril water on the tube containing 50 mg of methicillin and maintaining at −20 °C.

Minimal inhibitory concentration (MIC) of methicillin has been assayed on LB broth, medium used to obtain planktonic aggregates in 96-well microliter plate format as reported^4^. Bacteria were inoculated into 200 µL of liquid growth medium in the presence of different concentrations of antibiotic. Initial optical (OD) density was 0.1. Growth was assessed after incubation for a defined period of time (15 h at 37 °C) by measuring every 5 minutes the OD at 600 nm (Clariostar absorbance reader: 180 cycles of 300 s; 200 s agitation at 200 rpm before each cycle). MIC corresponds to the lowest concentration of antibiotic that inhibited totally the visible growth of the bacterium, i.e. the concentration for which OD did not increase.

### Evaluation of the combined effect of methicillin and *trans*(NO, OH)-[RuFT(Cl)(OH)NO](PF_6_) complex

Bacteria were treated with *trans*(NO, OH)-[RuFT(Cl)(OH)NO](PF_6_) and the solutions were irradiated. They were then grown as described above in 96-well microliter plate containing 200 μL of medium with different concentrations of methicillin. Growth was assessed for a defined period of time (15 h at 37 °C) by measuring the OD at 600 nm on the Clariostar absorbance reader.

### Quantification of the bactericidal effect of irradiated solution of *trans*(NO, OH)-[RuFT(Cl)(OH)NO](PF_6_)

*trans*(NO, OH)-[RuFT(Cl)(OH)NO](PF_6_) solutions of 0.1 µM concentration were prepared in LB in tubes and then irradiated for 10 min with the Hg lamp (32 mW). Bacteria were then inoculated in these tubes. 5 µg/mL of methicillin (corresponding to the MIC in presence of irradiated *trans*(NO, OH)-[RuFT(Cl)(OH)NO](PF_6_) were added to the tubes. Suspensions of bacteria were then grown on the surface of agar plate for 24 h and counted.

### Quantification of the bactericidal effect of nitrate and nitrite ions

NaNO_2_ and NaNO_3_ solutions of 0.01 µM were prepared in LB in tubes where bacteria were then inoculated. 5 µg/mL of methicillin (corresponding to the MIC in presence of irradiated *trans*(NO, OH)-[RuFT(Cl)(OH)NO](PF_6_)) were added to the tubes. Suspensions of bacteria were then grown on the surface of agar plate for 24 h and counted.

## Supplementary information

Electronic supplementary information
