# Author Correction: Effect of *trans*(NO, OH)-[RuFT(Cl)(OH)NO](PF_6_) ruthenium nitrosyl complex on methicillin-resistant *Staphylococcus epidermidis*

**DOI:** 10.1038/s41598-019-48222-0

**Published:** 2019-10-17

**Authors:** Mathilde Bocé, Marine Tassé, Sonia Mallet-Ladeira, Flavien Pillet, Charlotte Da Silva, Patricia Vicendo, Pascal G. Lacroix, Isabelle Malfant, Marie-Pierre Rols

**Affiliations:** 1 Laboratoire de Chimie de Coordination du CNRS, 205 route de Narbonne, F-31077 Toulouse, France; 2 Institutde Pharmacologie et de Biologie Structurale, IPBS, Université de Toulouse, CNRS, UPS, Toulouse, France; 3 Laboratoire des Interactions Moléculaires et de la Réactivité Chimique et Photochimique, Université Paul Sabatier, 118 route de Narbonne, F-31062 Toulouse, France

Correction to: *Scientific Reports* 10.1038/s41598-019-41222-0, published online 19 March 2019

The Acknowledgements section in this Article is incomplete.

“We gratefully acknowledge the financial support provided by the French National Center for Scientific Research (CNRS), the University Paul Sabatier (COMUE) and the Région Midi-Pyrénées through the RuNOthérapie (grant to M.B.). We are grateful to Dr Jelena Kolosnjaj-Tabi for proofreading the manuscript.”

should read:

“We gratefully acknowledge the financial support provided by the French National Center for Scientific Research (CNRS), the University Paul Sabatier (COMUE) and the Région Midi-Pyrénées through the RuNOthérapie (grant to M.B.). We are grateful to Dr Jelena Kolosnjaj-Tabi for proofreading the manuscript. We are grateful to Dr Etienne Dague (LAAS, France) for helping F. Pillet with AFM measurements.”

