# Supplementary material for: Effect of trans(NO, OH)-[RuFT(Cl)(OH)NO](PF6) ruthenium nitrosyl complex on methicillin-resistant Staphylococcus epidermidis
Source: Sci Rep. 2019 Mar 19;9:4867. doi: 10.1038/s41598-019-41222-0 (PMC6424994; doi:10.1038/s41598-019-41222-0)
Supplement: Supplementary file 1 — Electronic supplementary information [file 41598_2019_41222_MOESM1_ESM.docx]

Effect of *trans*(NO, OH)-[RuFT(Cl)(OH)NO](PF_6_) ruthenium nitrosyl complex on methicillin-resistant *Staphylococcus epidermidis*

Mathilde Bocé ^a, b^, Marine Tassé ^a^, Sonia Mallet-Ladeira^a^, Flavien Pillet ^b^, Charlotte Da Silva ^b^, Patricia Vicendo ^c^, Pascal G. Lacroix ^a^, Isabelle Malfant *^a^, Marie-Pierre Rols ^b^

**Mass Spectrometry**


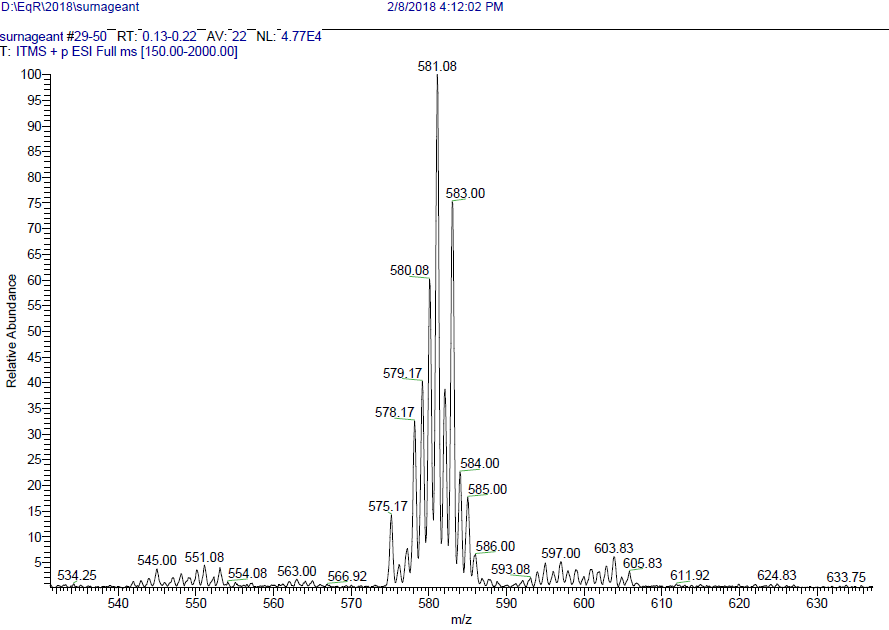


Fig. S1 Mass spectra of *trans* (NO, OH)-[RuFT(Cl)(OH)NO]^+^ in aqueous solution (0,5% DMSO)

**Crystallographic data for *trans*(NO, OH)-[RuFT(Cl)(OH)NO](PF_6_)**

Tab. S1 Crystal data and structure refinement for *trans*(NO, OH)-[RuFT(Cl)(OH)NO](PF_6_). 0.5 CH_3_CN, 2H_2_O.

Empirical formula C28 H20 Cl N4 O2 Ru, F6 P, 0.5(C2 H3 N), 2(H2 O)

Formula weight 782.53

Temperature 100(2) K

Wavelength 0.71073 Å

Crystal system, space group Triclinic, P -1

Unit cell dimensions a = 8.1415(6) Å alpha = 94.482(2) deg.

b = 11.4934(7) Å beta = 95.417(2) deg.

c = 16.4051(11) Å gamma = 97.154(2) deg.

Volume 1510.04(18) Å^3^

Z, Calculated density 2, 1.721 Mg/m^3^

Absorption coefficient 0.742 mm^-1^

F(000) 786

Crystal size 0.200 x 0.040 x 0.020 mm

Theta range for data collection 1.252 to 26.494 deg.

Limiting indices -10<=h<=10, -14<=k<=14, -20<=l<=20

Reflections collected / unique 48609 / 6221 [R(int) = 0.0692]

Completeness to theta = 25.242 99.9 %

Refinement method Full-matrix least-squares on F^2^

Data / restraints / parameters 6221 / 125 / 510

Goodness-of-fit on F^2 1.153

Final R indices [I>2sigma(I)] R1 = 0.0369, wR2 = 0.0926

R indices (all data) R1 = 0.0513, wR2 = 0.1042

Largest diff. peak and hole 0.741 and -0.640 e. Å^-3^


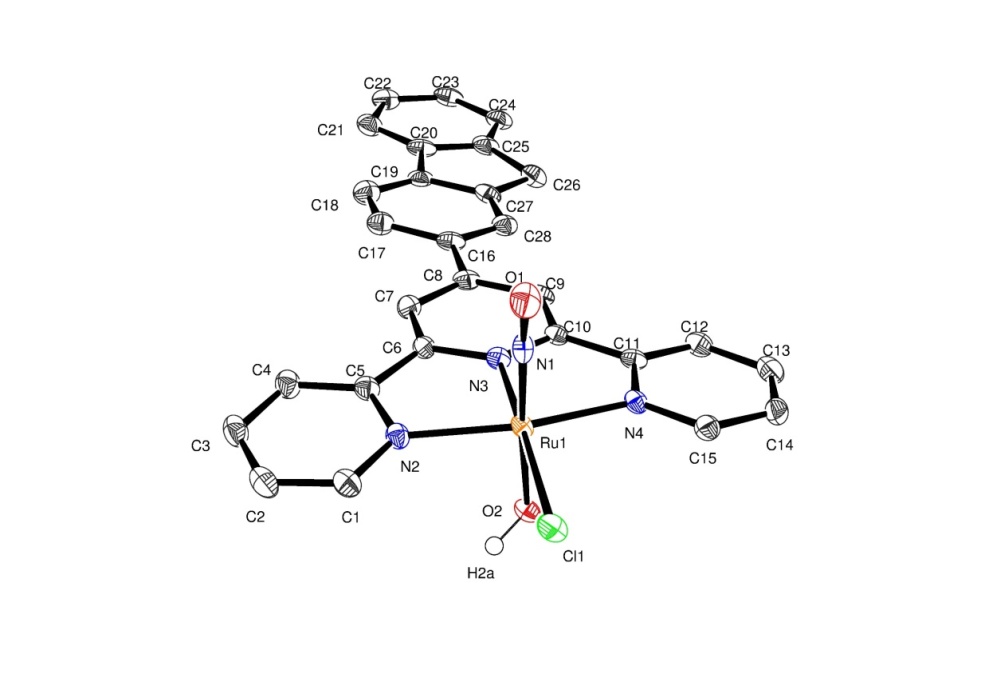


Fig. S2 *trans*(NO, OH)-[RuFT(Cl)(OH)NO]^+^ complex. Displacement ellipsoids are drawn at the 50%.probability level. Hydrogen atoms are omitted for clarity except H2a from OH ligand

Tab. S2 Atomic coordinates ( x 10^4^) and equivalent isotropic displacement parameters (Å^2^ x 10^3^) for *trans*(NO, OH)-[RuFT(Cl)(OH)NO](PF_6_). 0.5 CH_3_CN, 2H_2_O.

U(eq) is defined as one third of the trace of the orthogonalized Uij tensor.

_______________________________________________

x y z U(eq)

_______________________________________________

C(1) 5033(4) 3687(3) 1358(2) 22(1)

C(2) 3900(4) 3017(3) 1769(2) 26(1)

C(3) 3712(4) 3376(3) 2562(2) 25(1)

C(4) 4611(4) 4421(3) 2937(2) 22(1)

C(5) 5693(4) 5073(3) 2497(2) 19(1)

C(6) 6615(4) 6232(3) 2814(2) 17(1)

C(7) 6466(4) 6863(3) 3542(2) 19(1)

C(8) 7309(4) 8009(3) 3730(2) 18(1)

C(9) 8282(4) 8485(3) 3134(2) 18(1)

C(10) 8412(4) 7819(3) 2408(2) 16(1)

C(11) 9318(4) 8185(3) 1708(2) 17(1)

C(12) 10158(4) 9298(3) 1686(2) 21(1)

C(13) 10970(4) 9545(3) 999(2) 24(1)

C(14) 10924(4) 8692(3) 358(2) 23(1)

C(15) 10031(4) 7598(3) 403(2) 19(1)

C(16) 7136(4) 8709(3) 4506(2) 20(1)

C(17) 6251(4) 8196(3) 5119(2) 23(1)

C(18) 6008(5) 8842(3) 5830(2) 25(1)

C(19) 6675(4) 10016(3) 5950(2) 21(1)

C(20) 6648(4) 10923(3) 6639(2) 22(1)

C(21) 5915(4) 10836(3) 7381(2) 26(1)

C(22) 6118(4) 11844(3) 7936(2) 26(1)

C(23) 7006(4) 12876(3) 7749(2) 25(1)

C(24) 7699(4) 12960(3) 7010(2) 26(1)

C(25) 7507(4) 11971(3) 6461(2) 24(1)

C(26) 8111(4) 11827(3) 5613(2) 24(1)

C(27) 7562(4) 10542(3) 5359(2) 22(1)

C(28) 7791(4) 9894(3) 4638(2) 21(1)

Cl(1) 7573(1) 4791(1) -135(1) 22(1)

P(1) 8940(1) 2419(1) 3006(1) 29(1)

F(1) 10167(13) 2032(11) 2383(7) 48(3)

F(2) 7842(14) 1153(7) 2858(8) 39(2)

F(3) 7626(10) 2861(9) 3619(5) 49(2)

F(4) 9956(14) 3725(8) 3165(8) 47(3)

F(5) 9967(8) 2039(9) 3785(6) 59(3)

F(6) 7819(11) 2856(7) 2269(4) 36(2)

F(1') 9860(20) 2156(19) 2178(9) 47(4)

F(2') 7620(20) 1269(12) 2669(11) 40(3)

F(3') 8066(19) 2557(13) 3800(7) 49(3)

F(4') 10340(20) 3504(12) 3289(11) 46(4)

F(5') 10124(10) 1509(10) 3410(8) 45(3)

F(6') 7886(17) 3203(13) 2501(10) 52(3)

N(1) 9373(4) 5034(2) 1523(2) 18(1)

N(2) 5919(3) 4692(2) 1711(2) 18(1)

N(3) 7623(3) 6709(2) 2282(2) 16(1)

N(4) 9236(3) 7348(2) 1062(2) 16(1)

N(5) 3646(11) 5262(7) 4899(4) 45(2)

C(29) 2317(14) 5095(7) 4961(5) 42(2)

C(30) 509(13) 4946(15) 5059(11) 68(4)

O(1) 10441(3) 4490(2) 1653(2) 29(1)

O(2) 5940(3) 6668(2) 803(1) 18(1)

O(3) 6347(3) 8654(2) -2(2) 28(1)

O(4) 6213(4) 682(3) 1012(2) 40(1)

Ru(1) 7713(1) 5802(1) 1213(1) 14(1)

______________________________

Tab. S3 Bond lengths [Å] and angles [deg] for *trans*(NO, OH)-[RuFT(Cl)(OH)NO](PF_6_). 0.5 CH_3_CN, 2H_2_O.

_________________________________________________

Distances

C(1)-N(2) 1.341(4)

C(1)-C(2) 1.386(5)

C(1)-H(1) 0.9500

C(2)-C(3) 1.362(5)

C(2)-H(2) 0.9500

C(3)-C(4) 1.392(5)

C(3)-H(3) 0.9500

C(4)-C(5) 1.378(5)

C(4)-H(4) 0.9500

C(5)-N(2) 1.366(4)

C(5)-C(6) 1.475(5)

C(6)-N(3) 1.354(4)

C(6)-C(7) 1.372(5)

C(7)-C(8) 1.405(5)

C(7)-H(7) 0.9500

C(8)-C(9) 1.415(5)

C(8)-C(16) 1.479(5)

C(9)-C(10) 1.384(4)

C(9)-H(9) 0.9500

C(10)-N(3) 1.347(4)

C(10)-C(11) 1.483(5)

C(11)-N(4) 1.365(4)

C(11)-C(12) 1.377(5)

C(12)-C(13) 1.390(5)

C(12)-H(12) 0.9500

C(13)-C(14) 1.375(5)

C(13)-H(13) 0.9500

C(14)-C(15) 1.382(5)

C(14)-H(14) 0.9500

C(15)-N(4) 1.344(4)

C(15)-H(15) 0.9500

C(16)-C(28) 1.392(5)

C(16)-C(17) 1.415(5)

C(17)-C(18) 1.378(5)

C(17)-H(17) 0.9500

C(18)-C(19) 1.383(5)

C(18)-H(18) 0.9500

C(19)-C(27) 1.393(5)

C(19)-C(20) 1.479(5)

C(20)-C(25) 1.381(5)

C(20)-C(21) 1.411(5)

C(21)-C(22) 1.399(5)

C(21)-H(21) 0.9500

C(22)-C(23) 1.382(5)

C(22)-H(22) 0.9500

C(23)-C(24) 1.390(5)

C(23)-H(23) 0.9500

C(24)-C(25) 1.377(5)

C(24)-H(24) 0.9500

C(25)-C(26) 1.523(5)

C(26)-C(27) 1.503(5)

C(26)-H(26A) 0.9900

C(26)-H(26B) 0.9900

C(27)-C(28) 1.389(5)

C(28)-H(28) 0.9500

Cl(1)-Ru(1) 2.4020(8)

P(1)-F(3') 1.549(9)

P(1)-F(6') 1.553(10)

P(1)-F(1) 1.572(7)

P(1)-F(5) 1.580(5)

P(1)-F(4') 1.589(10)

P(1)-F(6) 1.596(6)

P(1)-F(2) 1.598(7)

P(1)-F(4) 1.611(7)

P(1)-F(2') 1.619(10)

P(1)-F(3) 1.635(7)

P(1)-F(1') 1.640(10)

P(1)-F(5') 1.643(7)

N(1)-O(1) 1.147(4)

N(1)-Ru(1) 1.761(3)

N(2)-Ru(1) 2.086(3)

N(3)-Ru(1) 1.981(3)

N(4)-Ru(1) 2.081(3)

N(5)-C(29) 1.090(12)

C(29)-C(30) 1.486(15)

C(30)-H(30A) 0.9800

C(30)-H(30B) 0.9800

C(30)-H(30C) 0.9800

O(2)-Ru(1) 1.950(2)

O(2)-H(2A) 0.830(10)

O(3)-H(3A) 0.841(10)

O(3)-H(3B) 0.843(10)

O(4)-H(4A) 0.851(10)

O(4)-H(4B) 0.843(10)

Angles

N(2)-C(1)-C(2) 122.0(3)

N(2)-C(1)-H(1) 119.0

C(2)-C(1)-H(1) 119.0

C(3)-C(2)-C(1) 118.9(3)

C(3)-C(2)-H(2) 120.5

C(1)-C(2)-H(2) 120.5

C(2)-C(3)-C(4) 120.0(3)

C(2)-C(3)-H(3) 120.0

C(4)-C(3)-H(3) 120.0

C(5)-C(4)-C(3) 119.0(3)

C(5)-C(4)-H(4) 120.5

C(3)-C(4)-H(4) 120.5

N(2)-C(5)-C(4) 120.9(3)

N(2)-C(5)-C(6) 115.6(3)

C(4)-C(5)-C(6) 123.4(3)

N(3)-C(6)-C(7) 119.5(3)

N(3)-C(6)-C(5) 113.5(3)

C(7)-C(6)-C(5) 126.9(3)

C(6)-C(7)-C(8) 120.7(3)

C(6)-C(7)-H(7) 119.6

C(8)-C(7)-H(7) 119.6

C(7)-C(8)-C(9) 117.4(3)

C(7)-C(8)-C(16) 121.3(3)

C(9)-C(8)-C(16) 121.3(3)

C(10)-C(9)-C(8) 120.3(3)

C(10)-C(9)-H(9) 119.8

C(8)-C(9)-H(9) 119.8

N(3)-C(10)-C(9) 119.2(3)

N(3)-C(10)-C(11) 113.1(3)

C(9)-C(10)-C(11) 127.7(3)

N(4)-C(11)-C(12) 121.2(3)

N(4)-C(11)-C(10) 115.5(3)

C(12)-C(11)-C(10) 123.3(3)

C(11)-C(12)-C(13) 118.5(3)

C(11)-C(12)-H(12) 120.8

C(13)-C(12)-H(12) 120.8

C(14)-C(13)-C(12) 120.2(3)

C(14)-C(13)-H(13) 119.9

C(12)-C(13)-H(13) 119.9

C(13)-C(14)-C(15) 118.9(3)

C(13)-C(14)-H(14) 120.5

C(15)-C(14)-H(14) 120.5

N(4)-C(15)-C(14) 121.5(3)

N(4)-C(15)-H(15) 119.3

C(14)-C(15)-H(15) 119.3

C(28)-C(16)-C(17) 118.5(3)

C(28)-C(16)-C(8) 120.8(3)

C(17)-C(16)-C(8) 120.6(3)

C(18)-C(17)-C(16) 121.7(3)

C(18)-C(17)-H(17) 119.1

C(16)-C(17)-H(17) 119.1

C(17)-C(18)-C(19) 118.8(3)

C(17)-C(18)-H(18) 120.6

C(19)-C(18)-H(18) 120.6

C(18)-C(19)-C(27) 120.7(3)

C(18)-C(19)-C(20) 131.6(3)

C(27)-C(19)-C(20) 107.7(3)

C(25)-C(20)-C(21) 121.1(3)

C(25)-C(20)-C(19) 109.0(3)

C(21)-C(20)-C(19) 129.9(3)

C(22)-C(21)-C(20) 117.5(3)

C(22)-C(21)-H(21) 121.2

C(20)-C(21)-H(21) 121.2

C(23)-C(22)-C(21) 120.2(3)

C(23)-C(22)-H(22) 119.9

C(21)-C(22)-H(22) 119.9

C(22)-C(23)-C(24) 122.0(3)

C(22)-C(23)-H(23) 119.0

C(24)-C(23)-H(23) 119.0

C(25)-C(24)-C(23) 118.2(4)

C(25)-C(24)-H(24) 120.9

C(23)-C(24)-H(24) 120.9

C(24)-C(25)-C(20) 121.0(3)

C(24)-C(25)-C(26) 128.9(3)

C(20)-C(25)-C(26) 110.1(3)

C(27)-C(26)-C(25) 102.1(3)

C(27)-C(26)-H(26A) 111.3

C(25)-C(26)-H(26A) 111.3

C(27)-C(26)-H(26B) 111.3

C(25)-C(26)-H(26B) 111.3

H(26A)-C(26)-H(26B) 109.2

C(28)-C(27)-C(19) 120.5(3)

C(28)-C(27)-C(26) 128.5(3)

C(19)-C(27)-C(26) 111.0(3)

C(27)-C(28)-C(16) 119.7(3)

C(27)-C(28)-H(28) 120.1

C(16)-C(28)-H(28) 120.1

F(3')-P(1)-F(6') 96.2(6)

F(1)-P(1)-F(5) 94.8(5)

F(3')-P(1)-F(4') 93.7(8)

F(6')-P(1)-F(4') 91.3(7)

F(1)-P(1)-F(6) 89.8(5)

F(5)-P(1)-F(6) 175.4(4)

F(1)-P(1)-F(2) 91.3(6)

F(5)-P(1)-F(2) 91.3(4)

F(6)-P(1)-F(2) 89.6(4)

F(1)-P(1)-F(4) 91.4(6)

F(5)-P(1)-F(4) 90.1(4)

F(6)-P(1)-F(4) 88.8(5)

F(2)-P(1)-F(4) 176.9(7)

F(3')-P(1)-F(2') 90.6(9)

F(6')-P(1)-F(2') 90.2(7)

F(4')-P(1)-F(2') 175.2(10)

F(1)-P(1)-F(3) 177.1(5)

F(5)-P(1)-F(3) 88.0(4)

F(6)-P(1)-F(3) 87.5(4)

F(2)-P(1)-F(3) 89.5(6)

F(4)-P(1)-F(3) 87.7(6)

F(3')-P(1)-F(1') 175.3(9)

F(6')-P(1)-F(1') 87.4(7)

F(4')-P(1)-F(1') 89.3(9)

F(2')-P(1)-F(1') 86.3(9)

F(3')-P(1)-F(5') 91.7(5)

F(6')-P(1)-F(5') 171.6(5)

F(4')-P(1)-F(5') 90.9(7)

F(2')-P(1)-F(5') 87.0(6)

F(1')-P(1)-F(5') 84.6(6)

O(1)-N(1)-Ru(1) 173.8(3)

C(1)-N(2)-C(5) 119.2(3)

C(1)-N(2)-Ru(1) 128.0(2)

C(5)-N(2)-Ru(1) 112.8(2)

C(10)-N(3)-C(6) 122.7(3)

C(10)-N(3)-Ru(1) 118.6(2)

C(6)-N(3)-Ru(1) 118.2(2)

C(15)-N(4)-C(11) 119.6(3)

C(15)-N(4)-Ru(1) 127.2(2)

C(11)-N(4)-Ru(1) 113.1(2)

N(5)-C(29)-C(30) 176.5(11)

C(29)-C(30)-H(30A) 109.5

C(29)-C(30)-H(30B) 109.5

H(30A)-C(30)-H(30B) 109.5

C(29)-C(30)-H(30C) 109.5

H(30A)-C(30)-H(30C) 109.5

H(30B)-C(30)-H(30C) 109.5

Ru(1)-O(2)-H(2A) 115(3)

H(3A)-O(3)-H(3B) 100(4)

H(4A)-O(4)-H(4B) 117(4)

N(1)-Ru(1)-O(2) 176.27(11)

N(1)-Ru(1)-N(3) 97.43(12)

O(2)-Ru(1)-N(3) 85.36(10)

N(1)-Ru(1)-N(4) 94.65(11)

O(2)-Ru(1)-N(4) 83.39(10)

N(3)-Ru(1)-N(4) 79.51(11)

N(1)-Ru(1)-N(2) 94.75(11)

O(2)-Ru(1)-N(2) 88.18(10)

N(3)-Ru(1)-N(2) 79.64(11)

N(4)-Ru(1)-N(2) 158.06(11)

N(1)-Ru(1)-Cl(1) 88.47(9)

O(2)-Ru(1)-Cl(1) 88.76(7)

N(3)-Ru(1)-Cl(1) 174.08(8)

N(4)-Ru(1)-Cl(1) 100.55(8)

N(2)-Ru(1)-Cl(1) 99.48(8)

_____________________________________________________________

Tab. S4 Anisotropic displacement parameters (A^2 x 10^3) for *trans*(NO, OH)-[RuFT(Cl)(OH)NO](PF_6_). 0.5 CH_3_CN, 2H_2_O.

The anisotropic displacement factor exponent takes the form:

-2 pi^2 [ h^2 a*^2 U11 + ... + 2 h k a* b* U12 ]

_____________________________________________

U11 U22 U33 U23 U13 U12

______________________________________________

C(1) 20(2) 20(2) 25(2) -1(1) 1(2) 1(1)

C(2) 18(2) 21(2) 38(2) 2(2) 0(2) 0(1)

C(3) 16(2) 24(2) 37(2) 8(2) 5(2) 2(2)

C(4) 18(2) 25(2) 25(2) 4(2) 6(2) 3(1)

C(5) 14(2) 20(2) 21(2) 2(1) 2(1) 5(1)

C(6) 12(2) 21(2) 18(2) 3(1) 3(1) 4(1)

C(7) 15(2) 24(2) 18(2) 3(1) 5(1) 5(1)

C(8) 13(2) 25(2) 19(2) 1(1) 0(1) 10(1)

C(9) 14(2) 17(2) 23(2) -4(1) -4(1) 4(1)

C(10) 12(2) 18(2) 19(2) 1(1) 0(1) 4(1)

C(11) 11(2) 20(2) 20(2) 0(1) -1(1) 7(1)

C(12) 18(2) 19(2) 24(2) -1(1) 0(1) 4(1)

C(13) 18(2) 21(2) 34(2) 5(2) -1(2) 0(1)

C(14) 16(2) 29(2) 25(2) 10(2) 4(2) 2(2)

C(15) 17(2) 21(2) 20(2) 2(1) 2(1) 3(1)

C(16) 15(2) 24(2) 22(2) -1(1) -3(1) 10(1)

C(17) 21(2) 26(2) 22(2) 1(1) 1(2) 6(2)

C(18) 23(2) 29(2) 24(2) 2(2) 3(2) 7(2)

C(19) 12(2) 33(2) 19(2) 4(2) -2(1) 7(2)

C(20) 16(2) 31(2) 20(2) -6(2) -3(1) 12(2)

C(21) 18(2) 30(2) 31(2) 4(2) -1(2) 5(2)

C(22) 18(2) 43(2) 18(2) -1(2) 4(1) 13(2)

C(23) 19(2) 31(2) 25(2) -9(2) -5(2) 11(2)

C(24) 15(2) 37(2) 26(2) 0(2) -2(2) 5(2)

C(25) 12(2) 35(2) 26(2) 1(2) -1(1) 9(2)

C(26) 17(2) 28(2) 26(2) -2(2) 1(2) 2(2)

C(27) 13(2) 24(2) 28(2) 0(2) -3(1) 5(1)

C(28) 15(2) 30(2) 20(2) 2(1) 1(1) 7(1)

Cl(1) 17(1) 26(1) 21(1) -5(1) 3(1) 2(1)

P(1) 22(1) 28(1) 36(1) 9(1) -1(1) -2(1)

F(1) 19(3) 28(4) 96(6) -17(4) 16(4) 2(2)

F(2) 35(4) 24(3) 56(6) 18(3) -9(3) -3(2)

F(3) 43(4) 65(5) 38(3) 7(3) 15(3) -6(3)

F(4) 55(5) 40(3) 40(4) -4(3) 10(3) -14(3)

F(5) 43(3) 66(5) 63(4) 31(4) -26(3) -10(3)

F(6) 39(3) 31(3) 38(3) 10(2) -2(2) 7(2)

F(1') 37(7) 40(6) 65(6) 1(5) 16(6) 7(5)

F(2') 26(5) 45(6) 44(7) 17(4) -13(4) -11(5)

F(3') 60(7) 49(6) 39(5) 7(4) 21(5) -3(5)

F(4') 38(6) 50(6) 41(7) -21(5) 20(5) -25(5)

F(5') 35(4) 48(5) 53(5) 10(4) -3(4) 10(3)

F(6') 41(5) 50(6) 81(7) 32(5) 30(5) 30(5)

N(1) 20(2) 13(1) 21(1) 2(1) 8(1) 0(1)

N(2) 14(1) 17(1) 21(1) 3(1) 3(1) 3(1)

N(3) 14(1) 17(1) 17(1) 0(1) 1(1) 4(1)

N(4) 11(1) 16(1) 20(1) 2(1) 2(1) 3(1)

N(5) 63(5) 47(5) 25(4) 3(3) 21(4) -3(4)

C(29) 71(6) 24(4) 31(4) 2(3) 14(5) -1(4)

C(30) 65(7) 61(7) 66(7) -25(6) 13(10) -27(10)

O(1) 19(1) 33(2) 41(2) 14(1) 10(1) 13(1)

O(2) 13(1) 17(1) 22(1) -1(1) -1(1) 3(1)

O(3) 30(2) 22(1) 32(2) 6(1) 4(1) 4(1)

O(4) 44(2) 41(2) 33(2) -7(1) -10(2) 14(2)

Ru(1) 12(1) 14(1) 16(1) -2(1) 3(1) 2(1)

**Photochemistry**

Tab. S5 Conditions for the quantum yield determination at λ_irr_=365 nm (with λ_obs_=300 nm) for *trans*(NO, OH)-[RuFT(Cl)(OH)NO](PF_6_) complex in aqueous solution at 25°C

| I_0_ (mol.L^-1^.s^-1^) | 9.66.10^-6^ |
| --- | --- |
| [A]_0_ (mol.L^-1^) | 4.57.10^-5^ |
| [B]_end of reaction_ (mol.L^-1^) | 4.52.10^-5^ |
| $\text{ε}_{\text{A}}^{\text{irr}}$ (mol^-1^.L.cm^-1^) | 16333 |
| $\text{ε}_{\text{A}}^{\text{obs}}$ (mol^-1^.L.cm^-1^) | 19322 |
| $\text{ε}_{\text{B}}^{\text{irr}}$ (mol^-1^.L.cm^-1^) | 11790 |
| $\text{ε}_{\text{B}}^{\text{obs}}$ (mol^-1^.L.cm^-1^) | 21658 |


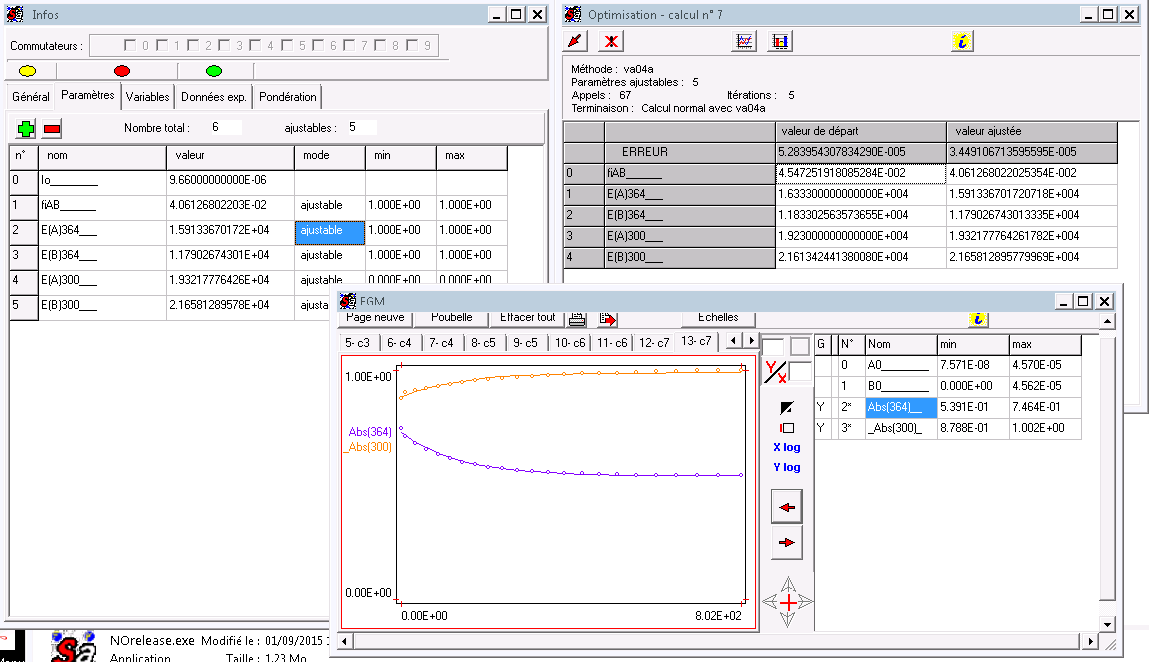


Fig. S3 Experimental (-) and calculated (○) absorbance under irradiation versus time (s) at 365 nm with λ_obs_ = 300 nm for *trans*(NO, OH)-[RuFT(Cl)(OH)NO](PF_6_) in aqueous solution (0.5% DMSO)

**Microbiology assays**


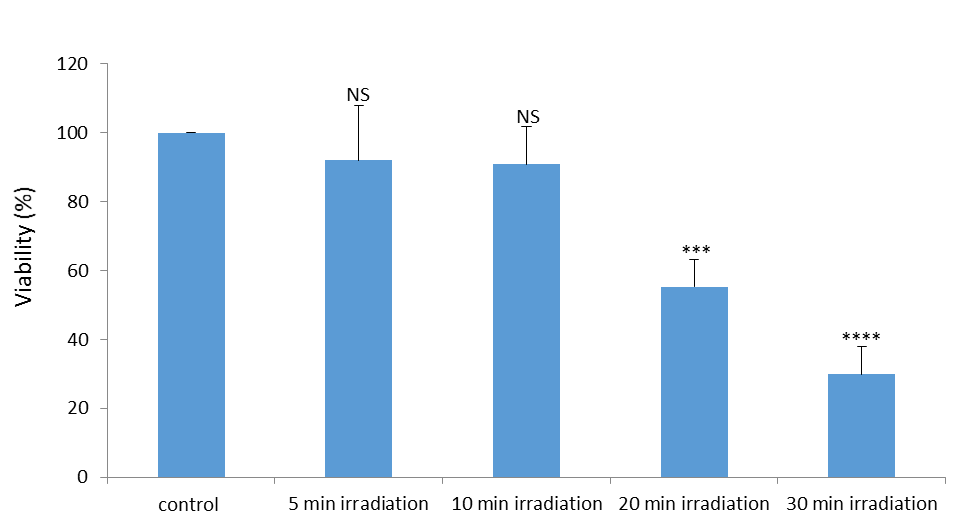


Fig. S4 Influence of the irradiation time on the ATCC 35984 viability

Fig. S5 Viability percentage of ATCC35984 (above) and ATCC 12228 (below) after treatment with *trans*(NO, OH)-[RuFT(Cl)(OH)NO](PF_6_), with or without irradiation. *: p-value, compared with untreated column (* : p < 0.05, ** : p < 0.01, *** : p < 0.005) ; Error bars: standard error.

Fig S6  Effect of methicillin and *trans*(NO, OH)-[RuFT(Cl)(OH)NO](PF_6_) on the growth of *Staphylococcus epidermidis* ATCC 12228 without /with irradiation of 0.1 µM [RuNO], [RuNO] stands for *trans*(NO, OH)-[RuFT(Cl)(OH)NO](PF_6_).

Tab. S6 Determination of MICs of methicillin in LB and MHB on *Staphylococcus epidermidis* ATCC 12228 and ATCC 35984 without/with 10 minutes irradiation of 0.1 µM *trans*(NO, OH)-[RuFT(Cl)(OH)NO](PF_6_). [RuNO] stands for *trans*(NO, OH)-[RuFT(Cl)(OH)NO](PF_6_).

|  | **Treatment** | **ATCC 12228** | **ATCC 35984** |
| --- | --- | --- | --- |
| **LB** | methicillin | **5 µg/mL** | **500 µg/mL** |
|  | **irradiated** [RuNO] with methicillin | **2 µg/mL** | **5 µg/mL** |
| **MHB, 2% NaCl (wt/vol)** | methicillin | **5 µg/mL** | **1 mg/mL** |
|  | **irradiated** [RuNO] with methicillin | **2 µg/mL** | **10 µg/mL** |

Fig. S7 Influence of the photoproduct, with or without methicillin, on the viability of ATCC 35984

Fig. S8 Influence of nitrites and nitrates with or without methicillin on the viability of ATCC 35984
